# Site-Selective Modification of (Oligo)Saccharides

**DOI:** 10.1021/acscatal.2c03876

**Published:** 2022-09-23

**Authors:** Martin D. Witte, Adriaan J. Minnaard

**Affiliations:** Stratingh Institute for Chemistry, University of Groningen, Nijenborgh 7, 9747 AG Groningen, The Netherlands

**Keywords:** site-selectivity, oligosaccharides, carbohydrates, late-stage functionalization, catalysis

## Abstract

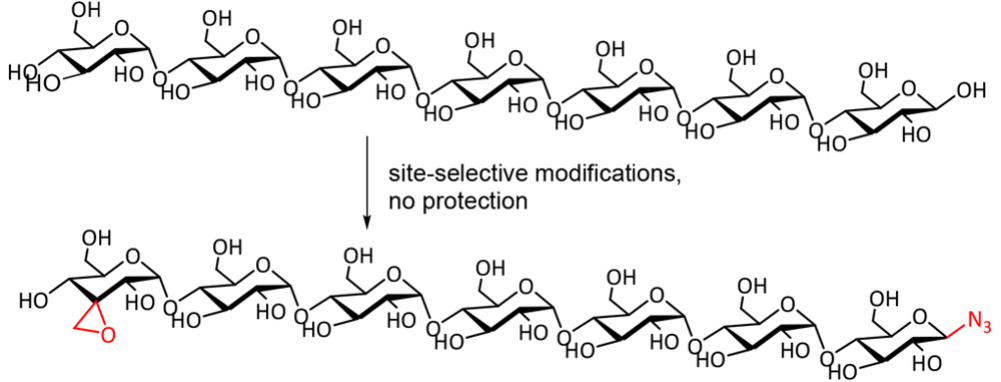

Oligosaccharides, either as such or as part of glycolipids, glycopeptides, or glycoproteins, are ubiquitous in nature and fulfill important roles in the living cell. Also in medicine and to some extent in materials, oligosaccharides play an important role. In order to study their function, modifying naturally occurring oligosaccharides, and building in reactive groups and reporter groups in oligosaccharides, are key strategies. The development of oligosaccharides as drugs, or vaccines, requires the introduction of subtle modifications in the structure of oligosaccharides to optimize efficacy and, in the case of antibiotics, circumvent bacterial resistance. Provided the natural oligosaccharide is available, site-selective modification is an attractive approach as total synthesis of the target is often very laborious. Researchers in catalysis areas, such as transition-metal catalysis, enzyme catalysis, organocatalysis, and photoredox catalysis, have made considerable progress in the development of site-selective and late-stage modification methods for mono- and oligosaccharides. It is foreseen that the fields of enzymatic modification of glycans and the chemical modification of (oligo)saccharides will approach and potentially meet each other, but there is a lot to learn and discover before this will be the case.

## Introduction

Being able to modify complex organic molecules in a selective way is one of the challenges in organic chemistry. In particular, in the field of natural products chemistry and in medicinal chemistry, many compounds containing multiple functional groups are more or less readily available. Instead of preparing analogues and derivatives of these by carrying out a novel synthesis from slightly altered building blocks, selective modification of the final product can be considerably more efficient. From a fundamental chemistry point of view, site-selective modification,^1^ or in a synthesis route; late-stage functionalization,^2^ is very interesting because it expands the knowledge we have of chemical reactivity and shows the white spots. Developing catalysts, reagents, and reaction conditions that discriminate among functional groups with very similar reactivity or activate seemingly unreactive C–H bonds is at the forefront of what we know and are able to do.^3^ We are currently not yet at the stage in which site-selective modification methods are available to prepare any product we would like to have. Most publications in the field of site-selective modification describe series of productive catalyst/substrate combinations, but catalyst design and product prediction are in their infancy.

Within the field of site-selective modification, carbohydrates form a relatively distinct class of substrates. This is because the selectivity challenge is dominated by the discrimination among the various hydroxy groups. Whereas the anomeric hydroxy group, in so-called “free” or “reducing” sugars, and the primary hydroxy group in a hexopyranoside can be relatively readily singled out, discrimination among the secondary hydroxy groups is achievable but very challenging. These hydroxy groups do have inherent reactivity differences, as is long known in the field of glycochemistry. In order to steer regio- and stereoselectivity in glycosidic bond formation between two monosaccharides, a large body of work has been devoted to the development of protecting group strategies that exploit these reactivity differences to single out a certain hydroxy group in the acceptor, and protect and activate the donor, so that the bond formation is both regio- and stereoselective.^4^

Approaching the situation from the opposite direction, that is, the selective modification of a hydroxy group in an unprotected glycoside, requires in part the same principles, namely using the subtle reactivity differences between the hydroxy groups, combined with neighboring group participation, chelation, and steric hindrance. Although some pioneering studies appeared already decades ago,^5^ with the development of many new synthesis and catalysis procedures, the field is facing rapid growth and the efforts have been reviewed several times. For a comprehensive and very thorough overview, the recent review of Dimakos and Taylor et al. in 2018^6^ is most instructive, and several overviews on more specific parts in this field have appeared as well.^7^ Therefore, this Perspective does not aim to comprehensively review the literature but rather reflects the opinion of the authors on the current state of the field, its embedding in carbohydrate chemistry in general, and the directions it might evolve. The term “unprotected carbohydrate” or “unprotected sugar” as often used in these studies needs some specification. Glucose as such is obviously an unprotected sugar. A glucose residue in the form of a glycoside, e.g., the anomeric hydroxy group is substituted, is still considered an unprotected sugar, also if the aglycon is simply methanol. When one or more of the hydroxy groups has been protected, it is more appropriate to speak of a partly or minimally protected sugar.

## Discussion

**Scheme 1:**
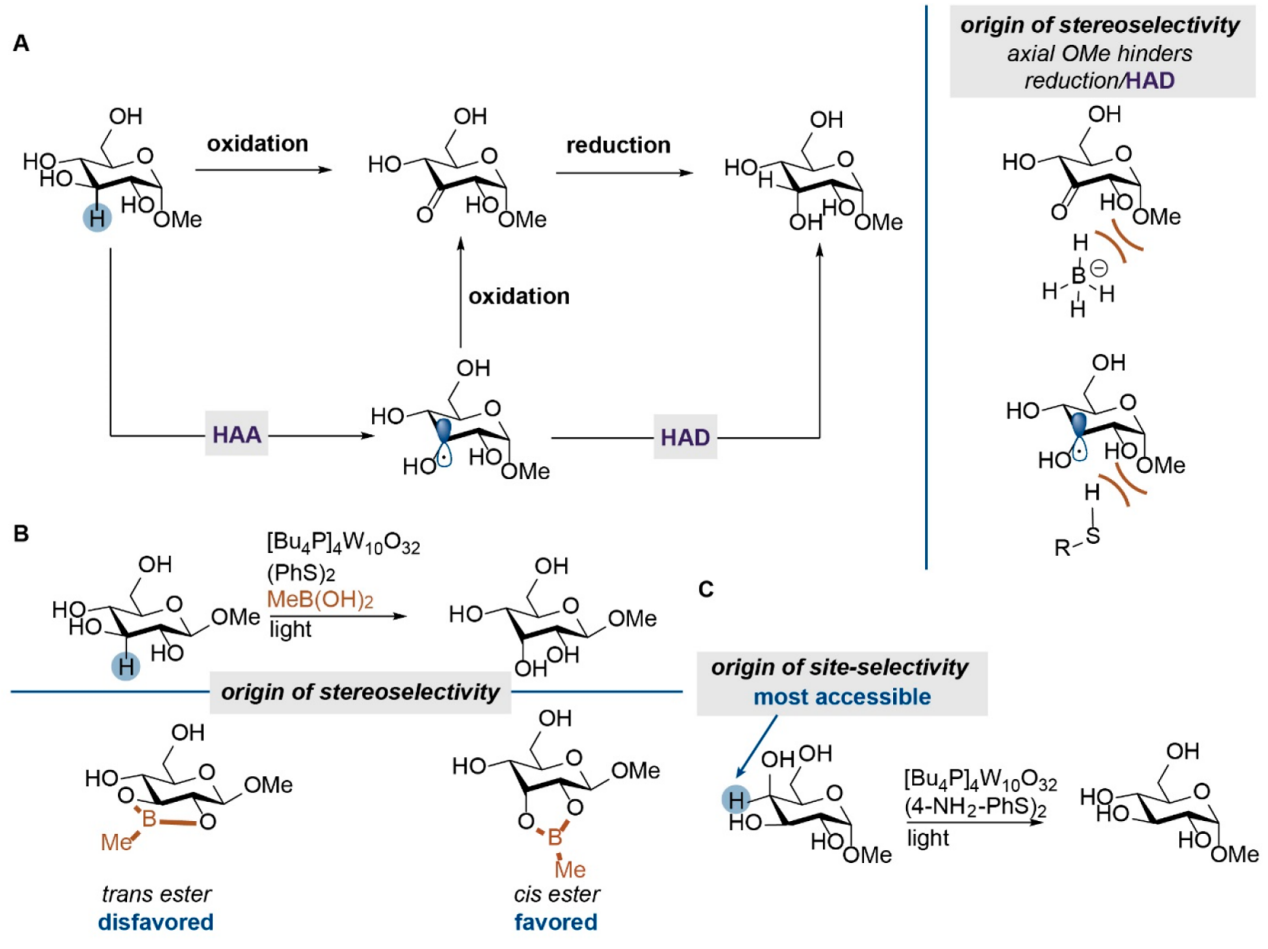
(A) Epimerization of the Low-Cost Monosaccharide Methyl-Glucose Leading to Methyl-Allose; (B) trans-to-cis Epimerization of β-Glucosides by Transient Trapping of the Epimerization Product; (C) cis-to-trans Epimerization of Galactosides Right panel: explanation of the stereoselectivity in the epimerization reactions. The blue spheres represent the hydride/hydrogen atom that is being abstracted.

There are several specific incentives to study the selective modification of carbohydrates. An important incentive can be to show the strength and the selectivity of a method, thereby leaving the potential application of the products to other researchers. An example in which both method development and application are important is the preparation of rare sugars from more commonly available ones. The physiological role of rare sugars is often not well understood, and rare does not mean less important, but in order to study these rare monosaccharides, availability is key. Rare sugars are often epimers of common monosaccharides and/or are deoxygenated on one or multiple positions. These rare sugars should therefore be accessible in only a few steps, provided that the desired stereocenter can be epimerized or deoxygenated selectively (for an example see Scheme 1A).

Traditionally, these reactions are either performed on suitably protected monosaccharides (for an overview of the methods to synthesize allose using protecting groups, see a recent review)^8^ or enzymatically. The use of enzymes in these epimerization reactions mostly requires the unprotected reducing sugars. There is a flourishing field of enzyme-catalyzed interconversions of monosaccharides, often catalyzed by isomerases, and some of these interconversions are carried out at scale (>1 kg).^9^ The processes are important mainly for food applications. It is troublesome, though, that most of these isomerization reactions are equilibria, and isolation of the product can therefore be challenging. The use of these enzymes in chemical carbohydrate synthesis is rare, also because the latter approach nearly invariably works with protected, organic soluble, substrates. Next to isomerases, a few oxidizing enzymes are well-known. In particular glucose oxidase and galactose oxidase are very versatile but have a strict substrate scope.^10^

Site-selective modification methods that allow the epimerization of hydroxy groups in the absence of protecting groups and with the same precision as enzymes, but without the strict substrate preferences, overcome important limitations of both chemical and enzymatic methods, in particular when applied on isolated oligosaccharides (*vide infra*). In addition, glycosylation reactions of the common monosaccharides are well described, so if modification can be effected once such a monosaccharide has been incorporated, this facilitates strongly the preparation of rare-sugar containing oligosaccharides.

Two approaches have evolved in catalysis to epimerize stereocenters in unprotected glucosides, namely sequential oxidation–reduction procedures and direct inversion procedures. These are illustrated for the conversion of methyl glucose into methyl allose (Scheme 1A, top part), although there are many more examples. The C3-hydroxy group of glucose can be oxidized regioselectively either using catalytic palladium/neocuproine in combination with oxygen or benzoquinone,^11^ or alternatively using a photoredox catalyst system in combination with oxygen.^12,13^ An axial anomeric substituent blocks axial attack of the reducing agent. Therefore, the subsequent reduction leads, in the case of α-glucose derivatives, to the corresponding allose analogues (Scheme 1A). Of note, an analogous transfer hydrogenation method has been developed to oxidize and epimerize hydroxy groups in aglycons. This method does not touch the carbohydrate (see the work of Hartwig et al.).^14^

An alternative to the sequential oxidation–reduction method is the direct inversion of the C3-hydroxy group. Elegant photoredox catalyst systems have been developed that epimerize the hydroxy group via hydrogen atom abstraction (HAA) and readdition using a hydrogen atom donor (HAD).^15^ With these systems, trans-configured glucosides can be isomerized into the less stable cis-configured allosides (Scheme 1A, bottom part). The first reported system uses quinuclidine in combination with 4-CzlPN and adamantane thiol as hydrogen atom donor to convert glucosides efficiently to allosides. The site-selectivity is determined in the hydrogen abstraction step. The quinuclidine radical preferentially abstract the hydrogen atom at the C-3 position of glucosides, as was also observed in photoalkylation reactions.^16^ The bulky hydrogen atom donor is likely essential to drive the trans-to-cis isomerization. As for the reductions of 3-ketoglucosides (*vide supra*), H-atom abstraction by the C3-centered radical will preferentially occur from the equatorial face, the axial face being blocked by the anomeric substituent (Scheme 1A). Blocking of the axial face has also been shown to determine the selectivities in photoalkylation reactions and deoxy-chlorination reactions.^16,17^ The same mechanism is likely determining the selectivity in the recently reported photoalkylation of myo-inositol.^18^ Recently, a second catalyst system was developed, which converts β-glucosides into β-allosides (Scheme 1B). This system uses the decatungstate anion and thiophenol catalyst pair. This sterically hindered decatungstate abstracts the most accessible H atom, whereas thiophenol in the hydrogen transfer reaction does not suffer from steric hindrance. To drive the trans-to-cis isomerization, the cis-diol has to be transiently trapped as the methyl boronate ester, which makes the cis-diol thermodynamically more favorable (Scheme 1B).^19^ The C3 position in α-glucosides, on the contrary, is not accessible for the decatungstate catalyst and therefore the C2 position is epimerized in these substrates. Moreover, in the absence of the boronate trapping agent, this catalyst system can be used to epimerize axial alcohols into equatorial alcohols, as was shown for galactosides (Scheme 1C).^20^ Finally, it was recently shown that direct epimerization of 1,2-trans to 1,2-cis diols in partly protected carbohydrates can be achieved with boron-mediated ruthenium-catalyzed hydrogen-borrowing/hydrogen transfer reactions, see the work of W. Tang et al.^21^ It will not be trivial, if possible at all, to dissect for all these reactions the kinetic and thermodynamic contribution to the observed regio- and stereoselectivity. To give an example, it has been shown by thorough computational studies using relativistic density functional theory that palladium-catalyzed oxidation in pyranoses preferentially takes place at C3 because the ring oxygen impedes the build-up of positive charge at C2 and C4 going to the transition state. The Δ*G* of the three keto-pyranoses differs only slightly.^22^ Still, upon oxidation at C4 mediated by a tin acetal, epimerization to the 3-ketopyranose readily happens. A different example is the epimerization of pyranoses in the work of MacMillan in which the intermediate cyclic boronate equilibrates to the thermodynamically favored product but after hydrolysis, this gives the overall “kinetic product”.^19^

The large majority of the reported site-selective modifications are carried out on glycosides, so with the anomeric hydroxy group substituted, because this blocks ring-opening (mutarotation), and the anomeric hydroxy group, in general, is quite reactive. Interestingly, both the palladium-catalyzed oxidation–sodium borohydride reduction sequence, and the photoredox approach have also been applied directly on α-glucose, so the reducing sugar.^23^ Besides epimerization methods, also a number of deoxygenating methods have been developed that can be used to prepare deoxygenated monosaccharides.^24^

**Scheme 2:**
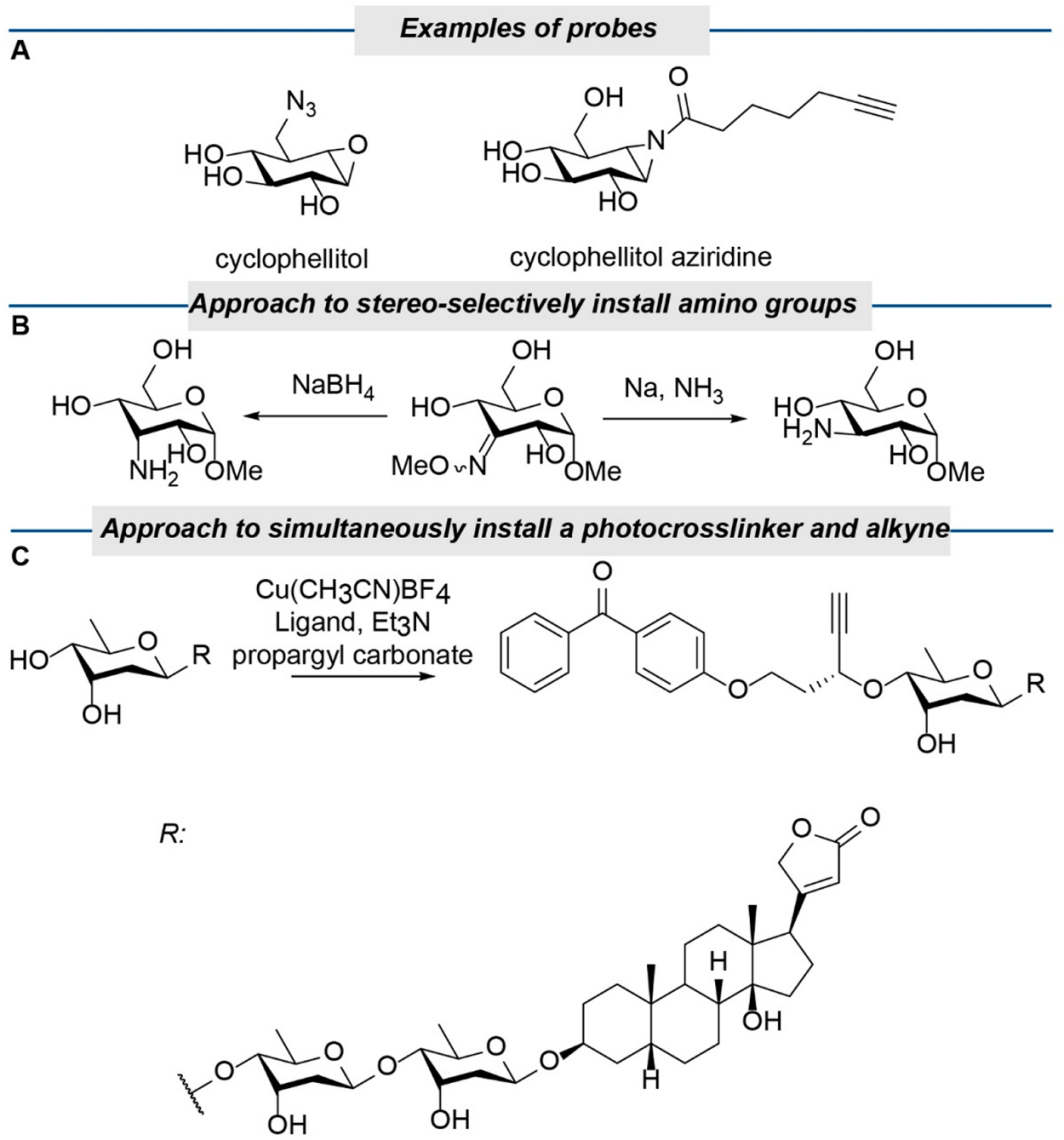
(A) Examples of Monosaccharides as Reactivity-Based Probes; (B) Diastereoselective Reduction Leading to Methyl 3-amino Glucose; (C) Single Step Conversion of Carbohydrates in Affinity-Based Probes by Site-Selective Propargylation

A second application for the site-selective modification of monosaccharides is the development of tool compounds that can be used to study the biological function of the carbohydrate. To facilitate these studies, the carbohydrates of interest have to be equipped with reactive groups, for activity-based probes for example, and with labels.^25^ In the case of a (latently) active group, the use of a protecting group strategy can complicate the synthesis, as orthogonality is required between the protecting groups and the active group. This can be a serious issue; frequently used reactive groups used in carbohydrate chemical biology are epoxides, (acyl)aziridines, azirines, alkynes, and azides (Scheme 2A). These functional groups are a challenge to combine with benzyl, CBz, and Boc protecting groups due to the conditions of the removal of the latter.^26^ Introducing these reactive groups in unprotected sugars can be a solution to this problem. The introduction of an azide functionality at a secondary hydroxy group in unprotected sugars via late-stage modification has rarely been reported, although the known sequence “site-selective oxidation-reductive amination” could be coupled to azide transfer. The stereochemistry of the amino group can be steered by the reduction method (Scheme 2B).^27^ Alternative, site-selective sulfonylation followed by a substitution reaction might fill this need.^28^ The introduction of an epoxide function in keto glucosides, as well as indium-mediated allylation and propargylation, have also shown to be effective methods to introduce chemical handles.^20^ Site-divergent *O*-propargylation of monosaccharides provides another means to site-selective introduce a bio-orthogonal handle. This latter method has proven to be very versatile, as the alkyne could be used in a later stage for the introduction of biotin and the antiviral agent zidovudine. Furthermore, a benzophenone functionalized reagent was developed that could be used to simultaneously introduce a photo-cross-linker as a bio-orthogonal handle, thus giving an affinity-based probe in a single step (Scheme 2C).^29^

Considering the current state of the art in regioselective or site-selective modification of carbohydrates, one can safely state that considerable progress has been made in recent years, and the field is growing very fast. The times when many groups in homogeneous catalysis would not consider carbohydrates as possible substrates and carbohydrate chemists would refrain from all too advanced catalysis methods are rapidly changing.^30^ The progress has become possible in part because of the growing knowledge about the subtle differences in reactivity of the different (secondary) hydroxy groups in a monosaccharide (mostly in its pyranoside form) together with the advent of transition metal catalysts that can use these subtle differences, either as such or in combination with agents that form transient chelates with the hydroxy groups altering their reactivity. The development of photoredox catalysis has strongly expanded the field,^31^ because carbohydrates have several advantages in this connection. The H–O bond of a hydroxy group is relatively stable to homolytic cleavage, so “protection” of hydroxy groups in a photochemical reaction is not a necessity. In addition, many photochemical reactions can be carried out in water or aqueous environment. This is an advantage considering the polar nature of unprotected sugars. Next to this, carbohydrates do not absorb UV/vis light. No doubt this suitability of carbohydrates for single electron processes will also make them applicable in electrosynthesis. There are several papers in the (older) literature that show this as a viable option^32^ but this field can be expanded considerably more.

Whereas it is beyond discussion that many selective transformations on unprotected monosaccharides are now established, this does not mean that the protocols are now the preferred way to make multigram amounts of product as starting material for further synthesis. The literature on the selective protection of monosaccharides followed by modification of the remaining hydroxy group is so advanced that it remains very difficult to surpass this with transformations on unprotected starting materials. This might be illustrated by the recently reported studies on the preparation of rare deoxy- and aminosugars. Wendlandt et al. showed in a beautiful study that the combination of photoredox catalysis and manganese-promoted radical migration leads to a variety of keto-deoxy monosaccharides, versatile starting materials for the synthesis of rare (amino) sugars.^24c^ Most of these compounds had been prepared earlier using protecting group strategies, and although the number of steps in the new photoredox approach was significantly less, the overall yield was not necessarily improved. A similar experience was reported earlier in the synthesis of aminosugars using palladium-catalyzed oxidation followed by reductive amination.^33^

Without a doubt, one of the main bottlenecks here is that unprotected carbohydrates are difficult to isolate and purify because of their high polarity. This is in particular the case for reducing sugars such as glucose, which “only” dissolves in water and dimethylsulfoxide. For di-, tri-, and oligosaccharides, this plainly is the case as well. For the corresponding glycosides without any lipophilic groups, the removal of salts or other water-soluble compounds is very difficult and the desired products do not readily elute from a silica column. This is reflected in literature; the procedures reported for the site-selective modification of monosaccharides are reported typically on mmol scale and provide “acceptable” but not excellent yields, and are not yet used to prepare the multigram amounts of starting material required for carbohydrate synthesis. The field would benefit from novel, preparative, methods for the purification of unprotected carbohydrates. Whereas on an analytical scale, High-Performance Liquid Chromatography (HPLC) methods based on polarity differences (Dionex, HILIC) and GC methods (using persilylation and pertrifluoroacetylation as derivatization methods) are well-established, the situation is less bright for straightforward preparative purification methods, except when crystallization can be used. Preparative HPLC is expensive and available only in specialized laboratories. “Bench-top” methods which are regularly used are column chromatography with diol-coated silica and dichloromethane/methanol or chloroform/methanol as the eluent. Alternatively, chelation chromatography (an ion exchange column charged with calcium or barium) or size exclusion chromatography can be used but the loading of these columns is limited. Classical “reverse phase” column chromatography using activated carbon as the stationary phase is occasionally applied using water/*t-*butanol as the eluent.^34^ The problem here is that pressure cannot be used readily as this blocks the column, and in addition “activated carbon” is not sufficiently defined to guarantee reproducible results in different laboratories.

Solving the purification issues may make site-selective modification of unprotected monosaccharides more main-stream and can lead in some cases to replacement of existing protecting group-based routes but for many substrates it will remain difficult to compete with the well-established routes that employ protecting groups or enzymatic modification. The most important power of the site-selective methods lies in our opinion therefore also not in monosaccharide modification, but in the possibility to manipulate di- and oligosaccharides.

The modification of monosaccharides, either with or without the use of protecting groups, is an active research field, but the challenges increase even more when we move from monosaccharides to di-, tri-, and even oligosaccharides.^35^ The modification of a single hydroxy group in members of these compound classes is very difficult. The rapidly increasing number of hydroxy groups simply does not allow a condensed all-but-one protection approach, and targeting one hydroxy group with a protecting group strategy is challenging as well. This often leads to the alternative; a bottom-up approach in which the oligosaccharide is prepared *ab initio* using the modified monosaccharide building blocks. This is a secure and reliable strategy but also comes with a large investment in effort and time. In particular, in cases in which the natural saccharide is available, a considerably more attractive scenario is site-selective, or late-stage, modification. Provided this can be done with sufficient fidelity, ánd the purification of the desired product is doable, this strongly facilitates the preparation of modified di-, tri-, and oligosaccharides. Two pioneering studies showed already that the site-selective modification of monosaccharide residues in the complex drugs erythromycin and vancomycin is possible.^36^

**Scheme 3:**
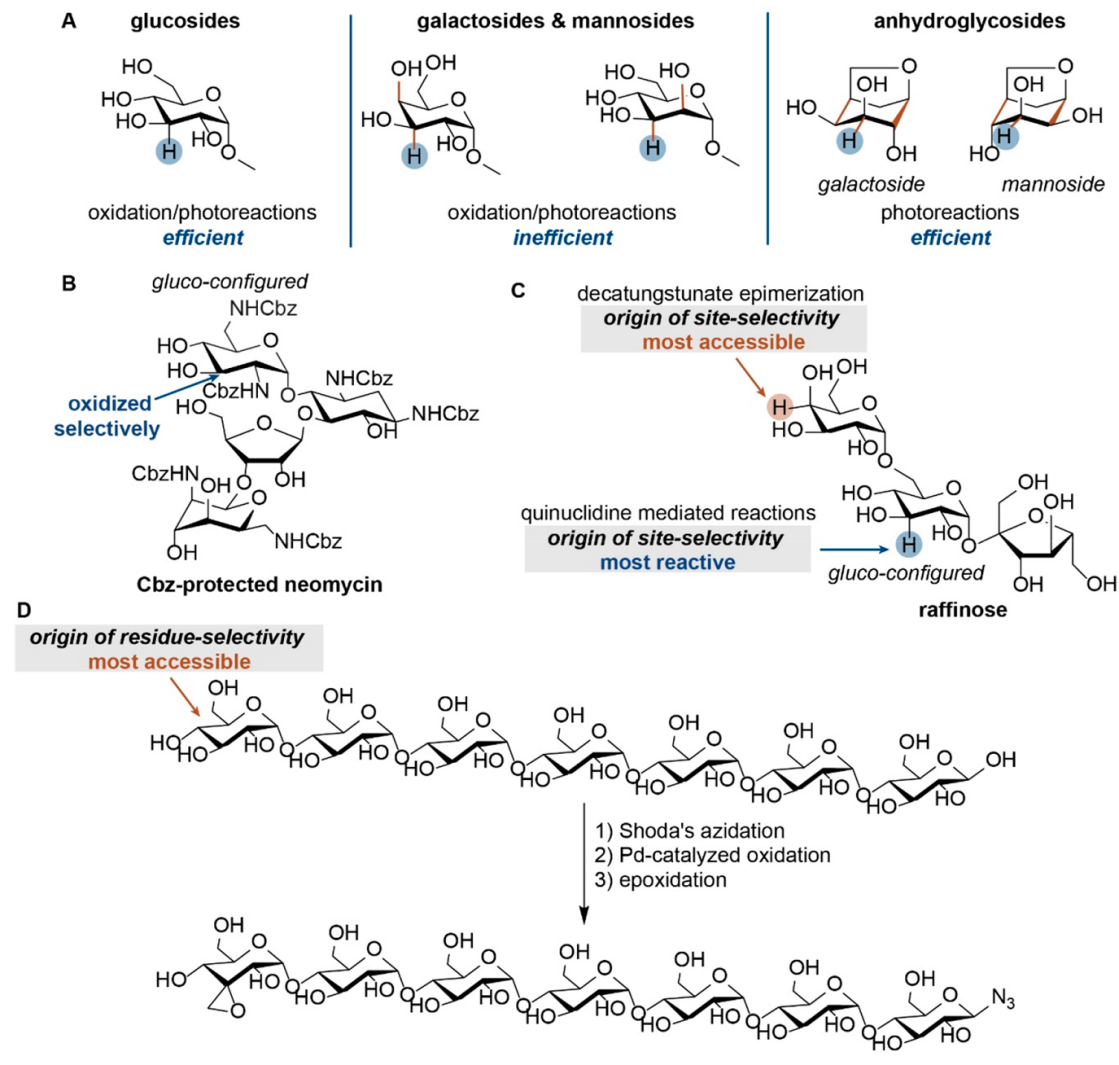
Site-Selective Oxidation in Oligosaccharides; (A) Reactivity Differences among Glucosides, Galactosides and mannosides; (B) Pd Catalyzed Oxidation of the Gluco-Configured Residue in Neomycin; (C) Site-Selectivities of Quinuclidine and Decatungstate Mediated Reactions in Raffinose; (D) Site-Selective Oxidation and Further Functionalization of Oligomaltosides The blue spheres represent the hydride/hydrogen atom that is being abstracted.

It is clear, though, that the site-selective modification of oligosaccharides is still in its infancy, although the first steps forward have been made in the past years. The reagent or catalyst used should not only discriminate between the different hydroxy groups in one saccharide unit but also discriminate between the different saccharide units in the substrate. This requires a somewhat different approach in the development of a site-selective method. Relative reaction rates become important, e.g., which monosaccharide is most reactive in a given reaction. Seemingly negative results in the monosaccharide series, substrates reacting only very slowly, become of key importance in the modification of oligosaccharides containing different units, which we illustrate below for two examples. For the palladium/neocuproine catalyzed oxidation reaction, we found in competition experiments that glucopyranosides can be oxidized with acceptable selectivity in the presence of manno- and galactopyranosides (Scheme 3A).^37^ We realized that these results suggest that gluco-configured units may be oxidized in glycans that also contain differently configured pyranosides and we recently exploited this in the selective oxidation of the antibiotics kanamycin and neomycin, a tri- and a tetrasaccharide, respectively (Scheme 3B).^38^ Admittedly, the amino groups in these substrates were protected. Of note, for an example in which selectively one of the amino groups in an aminoglycoside was modified, see the work of Bastian and Herrmann.^39^ Similarly, reactivity differences in photocatalytic systems can be used to selectively modify a residue within a di or oligosaccharide. From our initial study on photoalkylation, it was already clear that galactosides and mannosides react differently from glucosides (Scheme 3A).^40^ Since then, efforts have been directed to determine the reactivity differences of monosaccharides, including computational studies that determined the effect of the configuration on bond-dissociation energies.^41^ From the substrate scope-studies in these papers, it is apparent that quinuclidine-catalyzed H-atom abstraction proceeds preferentially on the C3-position of α-glucosides. Galactosides and mannosides do react only slowly at C3 and/or have an altered site-selectivity. Additives that chelate to a cis-diol can increase the reactivity, but lead to preferential H-atom abstraction at the carbon carrying the axial hydroxyl group.^42^ Interestingly, hydrogen atom abstraction on the C3-position is rather efficient when galactosides and mannosides are present in the corresponding 1,6 anhydro form, suggesting that the orientation of the hydroxy groups relative to the hydrogen that is being abstracted by the quinuclidine radical is of importance (Scheme 3A). The preference of the quinuclidine radical cation for α-glucosides can and has been exploited by the groups of Wendlandt, Taylor, and Liu and Wang to photochemically epimerize, oxidize, and alkylate the glucose residue in the disaccharide sucrose and the trisaccharide raffinose (Scheme 3C).^43^ The group of Wang even applied this to functionalize aminoglycosides. These two examples illustrate the importance of screening modification reactions on differently configured monosaccharides, and determining the relative reactivity of these substrates. Reactions that only proceed well on a particular substrate are ideal for the modification of oligosaccharides.

Nevertheless, this hierarchy in reactivity of monomeric sugars might not directly translate to oligosaccharides in which these monomers are present. Steric factors also play an important role in the selective modification of oligosaccharides, as is evident from our study on the oxidation of oligosaccharides (Scheme 3D).^33^ The reactivity difference in the oxidation of oligomaltoses by the palladium/neocuproine catalyst is solely based on steric effects in the entire oligosaccharides rather than reactivity difference within the monosaccharides. With high selectivity, the terminal residue in a series of oligomaltoses was oxidized at the C3 position, because the inherent reactivity of this position could be combined with the terminal residue being most accessible.

Apart from being a rather spectacular result, one hydroxy group out of 27 being selectively oxidized, the keto function in the product could be converted into an epoxide and in this way produced an affinity-based probe for maltose-binding protein.^25^ Very recently, an alternative approach to prepare oligomaltosides modified at the anomeric center and the terminal residue was reported. Full protection of cyclodextrins, followed by ring opening, provided an orthogonally substituted anomeric center and a free hydroxy group at C4 of the terminal residue. Provided the deprotection conditions are compatible with the active group introduced at C4, this is a versatile alternative.^44^

The importance of steric factors is further illustrated by the photochemical epimerization of raffinose. The decatungstate catalyst abstracts much more readily the accessible equatorial H atoms next to an axial hydroxy substituent. This preference enabled the epimerization of the C4-OH of the galactose residue in raffinose, yielding theanderose (Scheme 3C).^45^

From these findings, it is clear that model substrates are highly needed to probe the effect of sterics on different site-selective modification reactions and to better predict the outcome on a di/oligosaccharide. We have shown that THP-protected sugars may be used for these studies, provided that they do not react under the conditions used.^35^

**Scheme 4:**
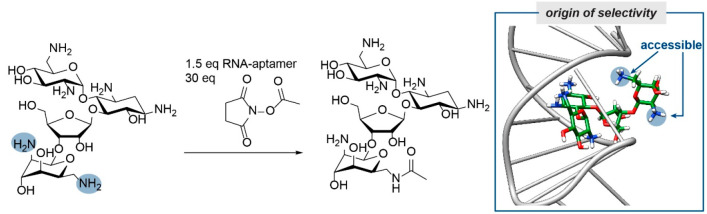
Site-Selective Amidation of Neomycin, Using RNA-Aptamers Representation of neomycin bound to the RNA aptamer was generated from PDB code: 1NEM with UCSF Chimera version 1.14.^47^

A third approach to induce site selectivity in oligosaccharides is to “cloak” the substrate in a supramolecular fashion. Cracks in this cloak should then allow site-selective modifications. Bastian and Herrmann used RNA-aptamers, which are negatively charged, to bind to the positively charged aminoglycoside neomycin. Subsequently, site-selective amidation was achieved (Scheme 4).^46^ Despite this success, scalability and finding the right “cloacking agent” is a serious issue here as in many supramolecular approaches. It remains to be seen whether this approach can be used on glycans as well. Nonetheless, this approach provides an elegant way to single out a reactive center and combining these supramolecular methods with the (photo)catalytic systems might increase the selectivity of the latter systems.

**Scheme 5:**
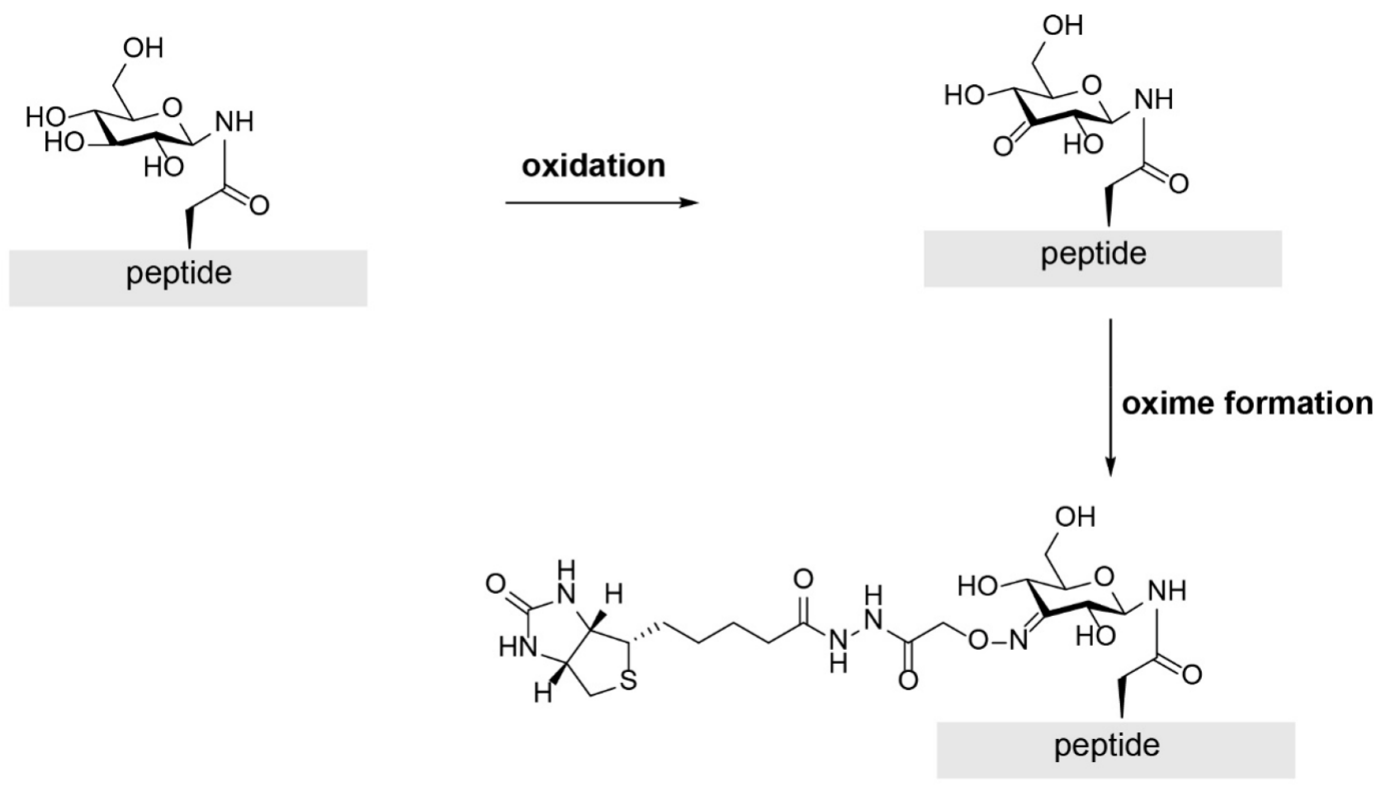
Site-Selective Oxidation of a Glycopeptide, Followed by Ligation with a Biotin Label Enables the Detection of Glucosylated Peptides

We foresee that the next leap from oligosaccharides will be the modification of glycans. From the viewpoint of (chemical) biology, being able to modify oligosaccharides is still largely an unmet need. Oligosaccharides function as antigens and recognition elements, and in addition to being present as such, many of these structures occur in conjugation with lipids (glycolipids), and peptides (glycopeptides) and connected to proteins (glycoproteins) and are referred to as glycans. It is well recognized that the application of chemical biology to these oligosaccharides, including glycans, broadens and deepens our knowledge of how a cell works. A potential next step is the application of this knowledge in medicine, in particular in the development of diagnostics and vaccines. The modification of oligosaccharides, composed of several different monosaccharide units in an endless array of possible connection modes, is already challenging, but this challenge becomes even bigger when changes have to be made in glycans because the aglycon (a lipid, peptide or protein) can interfere with the reaction. In particular proteins, and to a lesser extend peptides, are “reactive” in the sense that functional groups such as amino groups and thiols are more reactive than the hydroxy groups of the carbohydrate. It requires extensive research to develop chemical tools to selectively modify a certain carbohydrate residue in a glycan of a glycopeptide or protein. The first hesitating steps in this direction have been made.^48,49^ As an example, it turned out that glucose residues in several peptides could be oxidized with reasonable selectivity.^50^ The resulting ketone function was subsequently used to ligate the glycopeptide to biotin, for analysis by mass spectrometry. Specific fragments of a digested glycoprotein could in this way be identified (Scheme 5). The study showed the limitations of this method as well, because the palladium catalyst coordinated to the peptides and also serine and threonine residues were to some extent oxidized. A more advanced example comes from the work of Arnold, who used mutagenesis for the modification of the earlier mentioned galactose oxidase. The modified enzyme was able to oxidize galactose in some glycans selectively.^51^

**Scheme 6:**
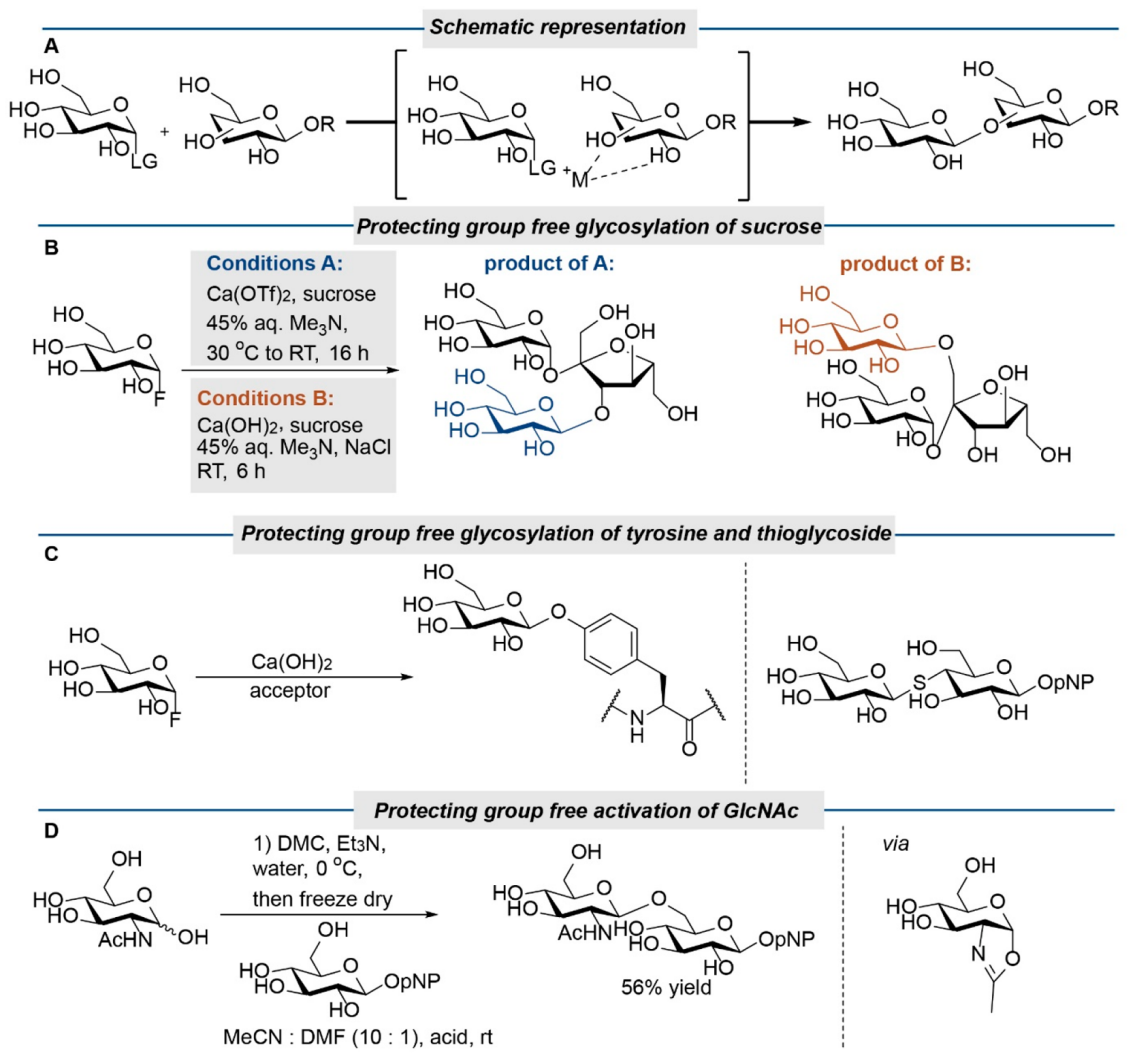
(A) Schematic Representation of Complexation-Induced Glycosidic Bond Forming Reactions with Unprotected Glycosides as Proposed by Miller and Schepartz; (B) Protecting Group Free Site-Selective Glycosylation of Sucrose; (C) Protecting Group Free Site-Selective Glycosylation of Peptides and Thioglycosides; (D) Protecting Group Free Glycosylation Procedure Developed by Fairbanks

A final research area in which we foresee that site-selective modification of unproctected carbohydrates will become more important is in the *de novo* synthesis of oligosaccharides. Glycosidic bond formation is at the heart of carbohydrate synthesis and performing this with partly protected monosaccharides is both challenging and important. There is considerable progress in the use of partially protected monosaccharides in combination with additives such as boronic acids, borinic acids and organotin reagents.^52^ These additives are used either to temporarily protect hydroxyl groups or to selectively activate hydroxy groups. This can be viewed as a special case of site-selective modification. In principle, two versions of this approach can be distinguished, one in which either the substrate (the acceptor) or the donor is unprotected, but the other reaction partner is protected, and one in which both are unprotected. While glycosylation with partly protected donors and acceptors has been studied in some detail, protecting group free glycosylation is an era that has just started. Only a few papers describe the latter approach. Key to a successful glycosidic bond formation between unprotected donors and acceptors is that the donor is only moderately electrophilic. Reactive donors will rapidly hydrolyze under aqueous conditions and will have a poor regioselectivity. Simultaneously, the acceptor should be a good nucleophile to facilitate the substitution reaction. Since alcohols are relatively poor nucleophiles, additives are needed to activate the hydroxy group that needs be modified. Ideally, the same additives can also activate the donor, which could allow complexation-induced glycosylation (Scheme 6A). This is nicely illustrated by the work of Miller, Schepartz, and co-workers.^53^ They demonstrated that glycosyl fluorides are suitable donors for the protecting group free glycosylation of sucrose derivatives. In the presence of calcium triflate and trimethylamine as a base, sucrose could be site-selectively glycosylated (Scheme 6B). The selectivity derived from an intricate hydrogen bonding network in the acceptor and could be tuned by changing the reaction conditions. This approach was recently expanded to glycosylate tyrosine residues in peptides (Scheme 6C). Using glycosyl fluoride as donors and Ca(OH)_2_ as a promotor, tyrosine residues in the peptides glucagon, endomorphin-2, and leu-enkephalin could be modified efficiently.^54^ Under these conditions, thiols also react with glycosyl fluorides and this has been used in the protection group free synthesis of thioglycosides and thioglycopeptides (Scheme 6C).^55^ In a very recent and beautiful paper, Fairbanks et al.^56^ showed that other activation methods also can be used to connect two unprotected monosaccharides (Scheme 6D). GlcNAc derivatives can be smoothly converted into oxazolines with DMC and triethylamine. Activation of the donor with p-toluenesulfonic acid allowed glycosylation of various monosaccharides. As the C6 hydroxy group of the acceptor is used, the problem to distinguish between all the hydroxy groups is at least largely avoided. Eventual regioselectivity issues are further alleviated by using an excess of acceptor (5 equiv compared to the donor). At the moment, the methods to connect unprotected monosaccharides are still highly substrate dependent. For example, the method of Fairbanks can only be applied using GlcNAc donors and the method of Miller and Schepartz only works for sucrose acceptors. However, lessons learned from the partly protected monosaccharides, such as organocatalysts like in a recent contribution from Wendlandt and Jacobsen et al.,^57^ that allow discriminating between the secondary hydroxy groups in the acceptor can eventually be translated to the fully unprotected carbohydrates.^58^

## Conclusions

In conclusion, site-selective modification of carbohydrates mainly originates from the field of homogeneous catalysis and to some extent from supramolecular chemistry. The enzymatic modification of oligosaccharides originates from biotechnology and biomolecular chemistry. These fields are now developing to reach the same goal, site-selective protective group free modification of saccharides in complex biomolecules. All these fields will have to contribute to reach these goals mentioned, and as is often the case, the biggest steps might result from a combination of these powerful tools. As major directions in the research on site-selective modification of oligosaccharides, we like to highlight the potential to modify these in the presence of amino groups, like in aminoglycosides, and thiols, such as in peptides and proteins. A second challenge is the chemical glycosylation of (largely) unprotected carbohydrates. It would be great if a bridge could be built between this approach and enzymatic glycosidic bond formation.
